# Proliferative Responses of Harbor Seal (*Phoca vitulina*) T Lymphocytes to Model Marine Pollutants

**DOI:** 10.1080/10446670310001593523

**Published:** 2002-12

**Authors:** Jennifer C. C. Neale, Judith A. Van de Water, James T. Harvey, Ronald S. Tjeerdema, M. Eric Gershwin

**Affiliations:** 1 Department of Environmental Toxicology University of California, Davis One Shields Avenue Davis CA 95616 USA; 2 Division of Rheumatology Allergy and Clinical Immunology TB 192, One Shields Avenue Davis CA 95616 USA; 3 Moss Landing Marine Laboratories California State University 8272 Moss Landing Road Moss Landing CA 95039 USA

## Abstract

In recent years, population declines related to viral outbreaks in marine mammals have been associated with polluted coastal waters and high tissue concentrations of certain persistent, lipophilic contaminants. Such observations suggest a contributing role of contaminant-induced suppression of cell-mediated immunity leading to decreased host resistance. Here, we assessed the effects of the prototypic polycyclic aromatic hydrocarbon (PAH), benzo[a]pyrene (B[a]P), and two polychlorinated biphenyls (PCBs), CB-156 and CB-80, on the T-cell proliferative response to mitogen in harbor seal peripheral lymphocytes. Despite the variability associated with our samples from free-ranging harbor seals, we observed a clear suppressive effect of B[a]P (10 uM) exposure on T cell mitogenesis. Exposures to 10 uM CB-156 and CB-80, and 1.0 and 0.1 uM B[a]P, did not produce significant depression in lymphoproliferation. Exposure to the model PAH at 10 uM resulted in a 61% (range 34-97%) average reduction in lymphoproliferation. We were able to rule out a direct cytotoxic effect of B[a]P, indicating that observed effects were due to altered T cell function. Based on our *in vitro* results, we hypothesize that extensive accumulation of PAH by top-trophic-level marine mammals could alter T cell activation *in vivo* and impaired cell-mediated immunity against viral pathogens.


*Developmental Immunology*, December 2002 Vol. 9 (4), pp. 215–221

Taylor & Francis healthsciences

### Proliferative Responses of Harbor Seal (*Phoca vitulina*) T Lymphocytes to Model Marine Pollutants

JENNIFER C.C. NEALE[a,]*, JUDITH A. VAN DE WATER[b], JAMES T. HARVEY[c], RONALD S. TJEERDEMA[a] and M. ERIC GERSHWIN[b]

[a]*Department of Environmental Toxicology, University of California, Davis, One Shields Avenue, Davis, CA, 95616, USA;* [b]*Division of Rheumatology, Allergy and Clinical Immunology, TB 192, One Shields Avenue, Davis, CA, 95616, USA;* [c]*Moss Landing Marine Laboratories, California State University, 8272 Moss Landing Road, Moss Landing, CA, 95039, USA*

In recent years, population declines related to viral outbreaks in marine mammals have been associated with polluted coastal waters and high tissue concentrations of certain persistent, lipophilic contaminants. Such observations suggest a contributing role of contaminant-induced suppression of cell-mediated immunity leading to decreased host resistance. Here, we assessed the effects of the prototypic polycyclic aromatic hydrocarbon (PAH), benzo[a]pyrene (B[a]P), and two polychlorinated biphenyls (PCBs), CB-156 and CB-80, on the T-cell proliferative response to mitogen in harbor seal peripheral lymphocytes. Despite the variability associated with our samples from free-ranging harbor seals, we observed a clear suppressive effect of B[a]P (10 uM) exposure on T cell mitogenesis. Exposures to 10 uM CB-156 and CB-80, and 1.0 and 0.1 uM B[a]P, did not produce significant depression in lymphoproliferation. Exposure to the model PAH at 10 uM resulted in a 61% (range 34–97%) average reduction in lymphoproliferation. We were able to rule out a direct cytotoxic effect of B[a]P, indicating that observed effects were due to altered T cell function. Based on our *in vitro* results, we hypothesize that extensive accumulation of PAH by top-trophic-level marine mammals could alter T cell activation *in vivo* and impaired cell-mediated immunity against viral pathogens.

*Keywords*: Benzo[a]pyrene; Harbor seal; Immunotoxicity; Lymphoproliferation; PAH; PCB

### INTRODUCTION

Understanding immune alterations related to environmental contamination has become an important priority in marine mammal research because of the high incidence in recent years of mass mortality events, unexplained population declines, and strandings among marine mammal populations (Dietz *et al.*, 1989; Marine Mammal Commission, 1999; Kennedy *et al.*, 2000). These events have been largely attributed to disease outbreaks caused by established or newly identified members of the genus *Morbillivirus* (Geraci *et al.*, 1982; Osterhaus and Vedder, 1988; Duignan *et al.*, 1995). Common features of these massive die-offs include highly polluted habitat and high tissue concentrations in affected individuals of persistent, lipophilic contaminants, including those known to have deleterious effects on the immune systems of rodents and humans (Hall *et al.*, 1992; Visser *et al.*, 1993; Reijnders, 1994). Whereas contaminant-induced immune suppression has been suggested as a major contributing factor in marine mammal disease epizootics, causal relationships and potential mechanisms of immunotoxicity have not been established (Olsson *et al.*, 1994; Marine Mammal Commission, 1999; van Loveren *et al.*, 2000).

The harbor seal (*Phoca vitulina*) is an ideal model organism for investigating contaminant-induced immune alterations in marine mammals. Fish-eating seals are long-lived, maintain large adipose deposits, and occupy high trophic levels in the marine food chain, thus accumulating relatively high levels of lipophilic contaminants (Boon *et al.*, 1987; Young *et al.*, 1998; Neale, unpublished data). Among marine mammals, harbor seals experience relatively high exposures to such chemicals, as they are non-migratory and frequent coastal habitats characterized by industrial centers, heavy marine traffic, and urban and agricultural runoff. Accordingly, Pacific harbor seals have been identified as one of four key indicators for coastal marine habitats by the California Department of Fish and Game. As an integrator of contamination of nearshore marine environments, the harbor seal also serves as a model for humans in communities dependent on consumption of seafood from polluted local waters.

Among the lipophilic chemicals that accumulate in marine mammal tissues are polycyclic aromatic hydrocarbons (PAHs), and polychlorinated biphenyls

*Corresponding author. Tel.: +1-530-752-2534/3286. Fax: +1-530-752-3394. E-mail: jcneale@ucdavis.edu

ISSN 1044-6672 print © 2002 Taylor & Francis Ltd
DOI: 10.1080/1044667031000159352

(PCBs). PAHs constitute a diverse class of ubiquitous industrial and environmental pollutants. Sources include forest fires and fossil fuel combustions, decay of organic materials, volcanic eruptions, and crude oil spills. PCBs are widespread anthropogenic pollutants that were manufactured in the U.S. until 1977 for use in electrical equipment. Decades after the ban on manufacture of these compounds, PCBs remain priority contaminants due to their great environmental persistence. Because PCBs and PAHs are bioconcentrated through the food chain, food sources represent the greatest exposure route for these top trophic level predators.

In this pilot study, we used a model PAH and two PCBs in an *in vitro* system to address the link between exposure to marine pollutants and lymphocyte function relevant to host resistance in the harbor seal. The cell-mediated arm of acquired immunity is most relevant to the mammalian immune response to viral pathogens. The proliferation of T cells in response to mitogens is one measure of T-cell function that is used in Tier I immunotoxicity tests *in vitro* and is frequently evaluated because of its correlation with *in vivo* host resistance (Luster *et al.*, 1988). This assay is considered a robust measure of the T-cell-mediated immune response to viral pathogens and of immunocompetence in general. We assessed the effects of the prototypic PAH, benzo[a]pyrene (B[a]P), a compound having both widespread occurrence in the environment and known immunotoxic effects in laboratory animals, on the T-cell proliferative response to mitogen of harbor seal peripheral lymphocytes (Ladics *et al.*, 1992; White *et al.*, 1994). For comparative purposes, two PCB congeners also were evaluated is this assay—CB-156 (2,3,3′,4,4′,5-hexachlorobiphenyl), a moderately chlorinated, mono-ortho PCB congener often present in biological samples, and CB-80 (3,3′,5,5′-tetrachlorobiphenyl), a less chlorinated, non-ortho congener.

### MATERIALS AND METHODS

### Samples

Seals were captured via land-grab and beach seine net techniques at haul-out sites in north-central California between May 2001 and February 2002 (Yochem *et al.*, 1987; Jeffries *et al.*, 1993; NMFS Scientific Research Permit No. 555–1565). Individuals were physically restrained and sex, mass, standard length (SL), and estimated age were recorded. A condition index (ratio of mass to SL) was calculated for each individual (Kopec and Harvey, 1995). Blood was drawn from the extradural venous sinus into sterile evacuated blood collection tubes containing acid citrate dextrose (Becton Dickinson Vacutainer Systems, Franklin Lakes, NJ, USA).

### Cell Preparation and Exposures

Peripheral blood mononuclear cells (PBMC) were isolated by centrifugation of blood for 20 min at 300*g*. Buffy coats were diluted 1:2 in RPMI 1640 medium (Sigma-Aldrich, St Louis, MO, USA). Mononuclear cells were further purified by density gradient centrifugation using 5 ml Histopaque-1077 (Sigma-Aldrich, St Louis, MO, USA) per 8.5 ml whole blood. Gradients were centrifuged for 30 min at 700*g*. The resulting white blood cell layer was removed and washed twice in Hank's Balanced Salt Solution (JRH Biosciences, Lenexa, KS, USA) with 10 min centrifugations at 200*g*. Cell viability was assessed via Trypan Blue solution (Sigma-Aldrich, St Louis, MO, USA) exclusion and viable cells were counted microscopically using a hemacytometer. Cell pellets were suspended in RPMI 1640 medium supplemented with L-glutamine (2 mM), gentamicin (0.08%), and fetal bovine serum (10%; hereafter referred to as "media").

B[a]P (Sigma-Aldrich, St Louis, MO, USA), and CB-156 and CB-80 (AccuStandard, New Haven, CT, USA) were suspended in anhydrous dimethylsulfoxide (DMSO; Sigma-Aldrich, St Louis, MO, USA) which also served as the vehicle control. Treatment solutions were diluted with media immediately before addition to wells. Final exposure concentrations in wells were 0.1, 1.0, and 10 uM for B[a]P and 10 uM for the vehicle and PCBs. A media control also was included.

### Mitogenesis

The proliferative response to mitogen by T lymphocytes was assessed using a standard blastogenesis assay (DiMolfetto-Landon *et al.*, 1995; Davila *et al.*, 1996). PBMC were cultured in 96-well microtiter plates (Corning, Incorporated, Corning, NY, USA) containing approximately $2 \times 10^5$ cells/well in a total volume of 0.2 ml. Cell cultures were incubated without mitogen or in the presence of phytohemaglutinin (Sigma-Aldrich, St Louis, MO, USA; 20 μg/ml, determined optimal concentration), a T-cell-specific mitogen in harbor seals (de Swart *et al.*, 1993). Cultures were incubated at 37°C in 5% $CO_2$ for 57 h before the addition of $^3$H-thymidine (spec. act. 6.7 Ci/mmol; PerkinElmer Life Sciences, Boston, MA), which was added at 1 μCi/well in 0.02 ml RPMI 1640. Following 15 additional hours of incubation, for a total incubation time of 72 h, cells were harvested onto filter paper using a Tomtech cell harvester (PerkinElmer, Boston, MA, USA). Filters were oven-dried and placed into bags with 11 ml of scintillation cocktail. Counts per minute (CPM) were determined by a Betaplate scintillation counter (PerkinElmer, Boston, MA, USA). Incorporation of radio-labeled thymidine represented DNA synthesis and cellular proliferation. All treatments and controls were performed in duplicate for mitogen-stimulated and unstimulated cultures for each individual. Background blanks were substracted from each sample CPM and measurements from duplicate wells were averaged before analysis. A stimulation index (SI) was calculated as the ratio of CPM in mitogen-stimulated cultures to that in unstimulated cultures. The reduction (%) in lymphoproliferation for B[a]P-treated cultures relative to media controls was calculated as: (1-(B[a]P SI/media SI)) $\times$ 100%.

### Cytotoxicity

Because the objective was to identify impacts of the model PAH and PCB toxic agents on cellular functioning, it was important to rule out the possibility of direct cytotoxicity. If B[a]P or PCBs had cytotoxic effects on T lymphocytes, we would expect to see reduced cell viability. To test for this effect, we examined viability of cells exposed to model agents vs. vehicle after 48-h incubation using duplicate lymphocyte cultures from 8 seals. Cell viability was assessed as described above.

### Data Analysis

Stimulation indices were log-transformed before all statistical tests, to meet assumptions of normality. To compare proliferative responses between cells treated with vehicle versus B[a]P/PCBs among seals, it was necessary to control for individual differences in baseline proliferation (indicated by media SI), which varied among seals and affected the extent of proliferative response when treated (and therefore detection of a suppressive effect of the model compound). Moreover, if B[a]P/PCBs but not the vehicle suppressed proliferation, we would expect to see an interaction between treatment effects and baseline proliferation due to systematically larger differences between the two treatments associated with higher baseline proliferation. We used a General Linear Model with "treatment" (i.e. 0.1, 1.0, and 10 uM B[a]P, 10 uM CB-156, 10 uM CB-80, and vehicle) SI as the factor and media control SI as a covariate. The treatment $\times$ media interaction term was used to indicate differences between the two treatments (i.e. differences between treatment vs. baseline slopes; test of homogeneity of regression (Tabachnick and Fidell, 1989)). The value of the slopes indicated the degree of suppression, with slope $= 1$ indicating no difference in proliferation between baseline and treated, and slope $< 1$ indicating a suppressive effect. Two-tailed *t*-tests were used to compare SI of males vs. females for agent-treated and vehicle-treated cultures. A two-tailed, paired samples *t*-test was used to compare the number of viable cells in agent-treated vs. vehicle-treated cultures. All statistical analyses were performed using SYSTAT (version 9, SPSS, Inc.) with an alpha of 0.05 for statistical significance.

### RESULTS

Blood samples were collected from 19 seals. Sample cultures not showing stimulation in response to mitogen ($n = 2$ seals) were excluded. The remaining 17 samples included 7 from San Francisco Bay, CA, collected 7/15/01–7/16/01, 5 from Elkhorn Slough (Monterey Bay, CA) collected on 5/18/01, and 5 from Pebble Beach (Monterey Bay, CA) collected on 5/16/01 and 2/7/02 (Table I). All animals appeared healthy at time of capture. Cells taken from all 17 individuals were used in the 10 uM B[a]P and CB-156 experiments. Due to limited sample volumes, however, only 9 seals were included in the experiments using CB-80 and lower concentrations (1.0 and 0.1 uM) of B[a]P.

A general trend of depression of lymphoproliferation was evident in all treatments relative to the vehicle control (Fig. 1), however the General Linear Model procedure failed to reveal significant effects of the two PCBs and lower concentrations of B[a]P (all $p > 0.7$). Therefore, the remainder of this discussion will focus on the experiment using 10 uM B[a]P.

TABLE I Sample characteristics for 17 seals, baseline (media controls) stimulation indices (SI), and reduction in proliferation (relative to baseline) due to exposure to the model PAH (10 uM B[a]P). Samples arranged in order of increasing baseline SI

| Capture site* | Sex | Age[†] | Mass (kg) | SL[‡] (cm) | Media SI | Reduction (%) |
|---|---|---|---|---|---|---|
| ES | F | P | 25 | 87 | 1.1 | No reduction |
| ES | M | P | 23 | 94 | 2.5 | No reduction |
| ES | M | P | 29 | 94 | 2.6 | No reduction |
| Bay | M | S | 37 | 111 | 3.2 | No reduction |
| PB | M | P | 27 | 86 | 3.3 | 56.0 |
| Bay | F | S | 37 | 123 | 6.0 | 43.5 |
| Bay | F | P | 20 | 89 | 6.4 | 34.1 |
| Bay | F | A | 50 | 122 | 6.5 | 56.3 |
| Bay | F | S | 44 | 117 | 8.3 | 37.8 |
| Bay | M | P | 26 | 96 | 9.3 | 55.7 |
| ES | M | S | 63 | 133 | 15.0 | 54.0 |
| Bay | F | S | 42 | 117 | 16.5 | 44.2 |
| ES | M | S | 58 | 131 | 22.1 | 60.5 |
| PB | F | P | 21 | 89 | 26.3 | 89.0 |
| PB | M | P | 27 | 90 | 29.8 | 73.7 |
| PB | F | P | 21 | 80 | 30.2 | 92.0 |
| PB | F | Y | 30 | 102 | 32.2 | 96.9 |

* Capture site: SFB, San Francisco Bay; ES, Elkhorn Slough; PB, Pebble Beach.
[†] Estimated age: P, pup; Y, yearling (1–2 years); S, subadult; A, adult.
[‡] Standard length (straight-line length measurement from tip of nose to tip of tail).

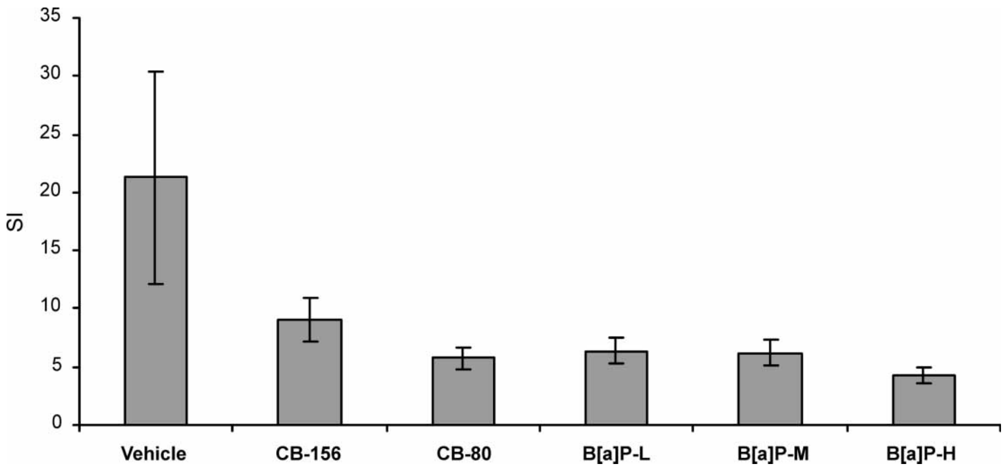

FIGURE 1 Lymphoproliferation of harbor seal T cells following *in vitro* exposure to vehicle (10 uM DMSO), CB-156 (10 uM in DMSO), CB-80 (10 uM in DMSO), and low (L; 0.1 uM), medium (M; 1.0 uM) and high (H; 10 uM) B[a]P. Data shown are means (± SE) of stimulation indices (SI; ratio of mitogen-stimulated CPM to unstimulated CPM) based on 17 harbor seals for vehicle, CB-156, and B[a]P-H, and 9 seals for CB-80, B[a]P-M, and B[a]P-L.

The General Linear Model procedure revealed a significant interaction between treatment (B[a]P or vehicle) SI and baseline (media) SI (Fig. 2; $F_{1,30} = 6.50, p = 0.02$), indicating significantly different relationships between the two treatments relative to media controls. The regression of SI in B[a]P-treated cultures relative to media controls produced a slope significantly and substantially less than 1 (slope estimate = 0.14, 95% CI: −0.15–0.43), indicating a clear suppressive effect of the model PAH. In contrast, the vehicle-media SI slope did not differ significantly from 1 (slope estimate = 0.85, 95% CI: 0.38–1.32), indicating no suppressive effect of the vehicle.

After stimulation by the mitogen, exposure to 10 uM B[a]P generally depressed the proliferative response, although large variation among individuals was apparent (Fig. 3). Because vehicle SI did not differ significantly from media SI and because there was less variance among media SI values, we calculated reduction in proliferation in B[a]P-treated cultures relative to media SI (baseline) for each individual. B[a]P-depressed proliferation of T lymphocytes in response to mitogen was evident for all individuals except those with the lowest baseline stimulation (Table I). The average reduction in lymphoproliferation due to exposure to the model PAH was 61% (range 34–97%). Because age was a potentially confounding factor, we also examined pups only (the only age class present at all 3 locations). When only pups were considered ($n = 6$), the depression of proliferation in B[a]P-treated cultures was even greater (67% reduction). Stimulation indices (log-transformed) did not differ significantly between sexes for B[a]P-treated ($p = 0.31$) or vehicle-treated ($p = 0.89$) cultures.

Small sample sizes and unequal representation of age classes across locations prevented the determination of independent effects of capture site and age. Because mass and standard length were necessarily highly correlated with age class, these also were not assessed as independent

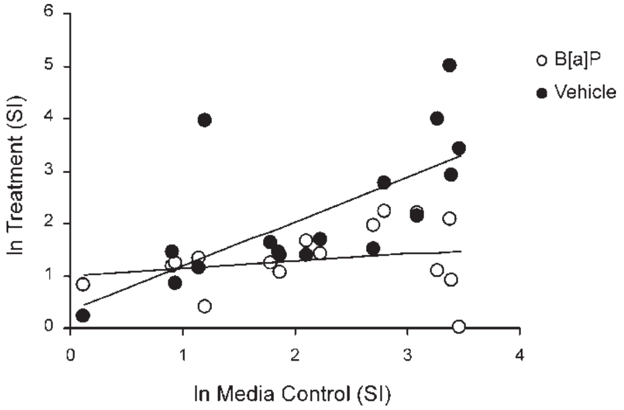

FIGURE 2 Stimulation indices (SI; ln-transformed) from 17 individual harbor seals for 10 uM vehicle- and B[a]P-treated cultures with linear regressions relative to baseline (media control) proliferation.

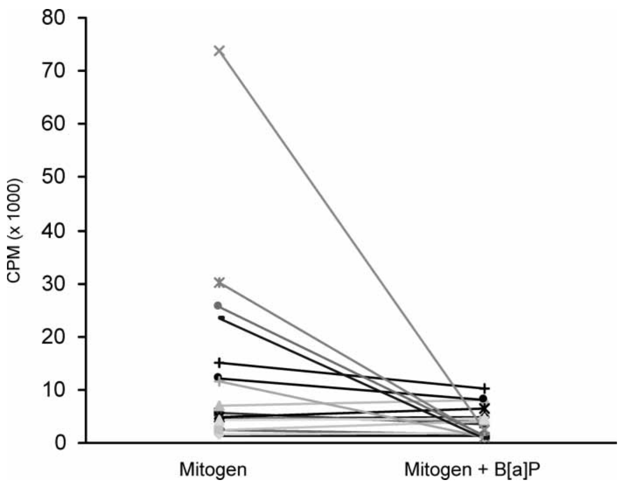

FIGURE 3 T cell proliferation in lymphocytes from 17 harbor seals. Cells were exposed *in vitro* to mitogen and vehicle only versus mitogen and 10 uM B[a]P.

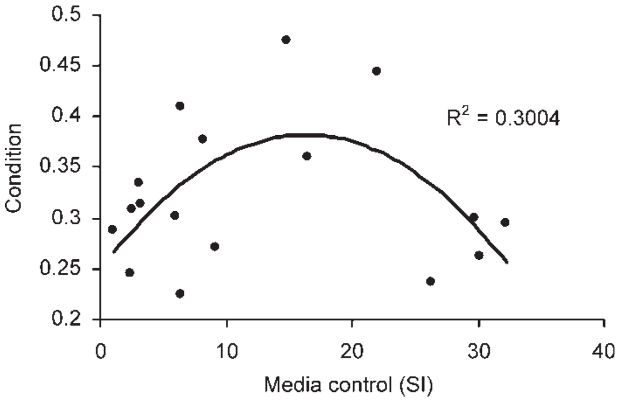

FIGURE 4 Relationship between physiological condition (indexed by the ratio of mass to standard length) and baseline proliferation (stimulation index for media control cultures) of T cells from 17 individual harbor seals. Data are fitted to a polynomial function.

factors. The relationship between physiological condition (based on our index) and baseline stimulation (media SI) was investigated and revealed a polynomial function (Fig. 4), indicating that animals in the greatest physiological condition showed moderate baseline stimulation. These same individuals also tended to be older and least affected by B[a]P treatment (i.e. showed highest SI for 10 uM B[a]P). Finally, viability in B[a]P-treated lymphocyte cultures did not differ from vehicle controls ($p = 0.39$), indicating that the observed effects of *in vitro* exposure to B[a]P were not caused by direct cytotoxicity.

### DISCUSSION

Increasing awareness of the widespread environmental occurrence and persistence of certain organic pollutants has generated great interest, among scientists as well as the general public, in their potential health effects on human and wildlife populations. Previous toxicity studies using laboratory animals indicate that the mammalian immune system is a sensitive target of persistent organic pollutants such as tetrachlorodibenzo-*p*-dioxin, PCBs, and PAHs (Tucker *et al.*, 1986; Ladics *et al.*, 1991; Stack *et al.*, 1999). Many reports have documented the specific suppressive effects of the carcinogenic PAHs on both humoral and cell-mediated immunity in rodents and humans (Ladics *et al.*, 1991; 1992; reviewed in White *et al.*, 1994; Davila *et al.*, 1996; Salas and Burchiel, 1998). Effects included decreased proliferation responses to T-cell mitogens as well as lymphoid cell death, lymphoid organ atrophy, altered lymphocyte subsets, apoptosis, decreased B-cell responses to mitogens and antigens, altered cytokine regulation, and changes in innate immune function. Despite evidence of widespread exposure of marine mammals to PAHs and their potential influence on health, and their recent identification as a research priority by the Marine Mammal Commission (1999), PAHs have received little attention in marine mammal research (Martineau *et al.*, 1994; Fossi *et al.*, 1997).

Here, we exposed harbor seal lymphocytes *in vitro* to model pollutants and examined the effects on one measure of cell-mediated immunity—the proliferation of T cells in response to mitogen stimulation. Although the *in vitro* approach utilized in this investigation represents an extreme reductionism relative to the very complex situation in the intact organism, it can provide insight into specific effects of model agents. Despite the variability associated with our samples from free-ranging harbor seals, we observed a clear suppressive effect of B[a]P exposure on T cell mitogenesis. The greatest effect on proliferation due to treatment with B[a]P was seen in cultures with relatively high stimulation responses; such samples may represent individuals whose peripheral lymphocytes were not already activated by immunotoxic or infectious agents. The lack of evidence of cytotoxicity indicates a direct effect of the model PAH on T cell function. Low statistical power may have decreased our ability to confirm depressive effects of the PCBs and lower concentrations of B[a]P.

Although studies such as the present one advance our understanding of possible immunotoxic effects of PAH at the cellular level, the mechanisms of PAH immunotoxicity are not yet fully understood (White *et al.*, 1994). In this study, only the parent compound was used and no attempt was made to determine if the immunosuppressive moiety was B[a]P or a metabolite(s). Various mechanisms of action of PAH immunotoxicity have been proposed, many of which involve formation of reactive metabolites via cytochrome P450 metabolism. Activation of B[a]P by P450-mediated metabolism to a dihydrodiol epoxide or radical cation metabolite occurs in splenic macrophages in the mouse (Ladics *et al.*, 1992). Human peripheral lymphocytes also possess cytochrome P450s, which metabolize PAHs (Davila *et al.*, 1995). Recent work indicates that several marine mammal species possess hepatic P450 enzymes with broadly similar activity to that of laboratory species (Hart *et al.*, 1996; Addison *et al.*, 1998; Stegeman *et al.*, 1998). It is reasonable to postulate that in the harbor seal, extrahepatic tissues such as peripheral blood leukocytes also may have this oxidative capacity. In this study, cell preparations contained macrophages in addition to B and T lymphocytes; these cell types were likely capable of biotransformation of B[a]P to metabolites which may have served as the proximate toxicant(s).

Previous investigations of contaminant-induced immune suppression in marine mammals have typically correlated impaired immune responses or less specific biomarkers with tissue levels of polyhalogenated aromatic hydrocarbons (PHAHs) such as the organochlorine pesticides and PCBs (Reijnders, 1994; Lahvis *et al.*, 1995; Simms *et al.*, 2000). While providing circumstantial evidence for immune suppression, causal relationships and mechanisms of action could not be established. Experimental research has been limited to studies of captive harbor seals fed environmentally contaminated fish, which indicated immune suppression related to PCBs

(Brouwer *et al.*, 1989; de Swart *et al.*, 1995; Ross *et al.*, 1996). However, presence and levels in fish of other potentially immunotoxic compounds such as metals and PAHs were not assessed.

Our work complements previous *in vivo* research by experimentally demonstrating a depressive effect of exposure to a model marine pollutant on an important lymphocyte function in cell-mediated immunity. The B[a]P exposure showing depressive effects *in vitro* in the present study (10 uM) is equivalent to 2.5 ppm, a concentration similar to that found in brain and liver (DNA adducts of B[a]P) of beluga whales of the St Lawrence Estuary (Quebec, Canada) and in liver (sum of 8 PAH) of South American fur seals of the Rio Grande, Brazil (Martineau *et al.*, 1994; Fossi *et al.*, 1997). Taken together, the data indicate that PAHs as well as PHAHs are immunotoxic marine contaminants with the potential to affect host resistance to disease in free-ranging pinnipeds and probably other marine mammals (and possibly human populations).

In conclusion, our findings indicate an immunosuppressive effect of the prototypic PAH on T-cell function and hence imply impairment of host resistance against viral pathogens *in vivo*. To our knowledge, this is the first study to investigate PAH exposure in harbor seals and directly demonstrate immunotoxic effects of a model marine pollutant on functional responses of lymphocytes in a marine mammal species. Future work in this area should include investigation of the possible effects of PAHs on other T-cell-mediated responses and attempt to illuminate the interactive effects of age, physiological condition, capture site (environment), and other potential factors at play in a free-ranging, outbred population.

### Acknowledgements

We thank B. Sacks for statistical advice and editorial comments; T. Mao, G. Nalbandian, T. Kenny, and E. Berg for general laboratory assistance; F. Gulland for veterinary expertise; and S. Oates, S. Allen, E. Grigg, D. Green, volunteers from Moss Landing Marine Laboratories and The Marine Mammal Center and the Richmond Bridge Harbor Seal Survey (funded by CalTrans) for assistance with seal capture. Funding support (to JCCN) included a Predoctoral Fellowship in Ecology (UCD), the Jastro-Shields Research Grant (UCD), a UC Marine Council Research Grant (#02 T CEQI 03 0104), and a NIH Traineeship in Environmental Toxicology (#5 T32 ES07059-25).

### References

Addison, R.F., Bullock, P., Lockhart, W.L. and Metner, D. (1998) "CYP1A concentrations, hepatic mono-oxygenase activities and organochlorine residue concentrations in beluga whales from NWT, Canada", *Marine Environmental Research* **46**, 121.

Boon, J.P., Reijnders, J.H., Dols, J., Wensvoort, P. and Hillebrand, M.T.J. (1987) "The kinetics of individual polychlorinated biphenyl congeners in female harbour seals (*Phoca vitulina*), with evidence for structure-related metabolism", *Aquatic Toxicology* **10**, 307–324.

Brouwer, A., Reijnders, P.J.H. and Koeman, J.H. (1989) "Polychlorinated biphenyl (PCB)-contaminated fish induces vitamin A and thyroid hormone deficiency in the common seal (*Phoca vitulina*)", *Aquatic Toxicology* **15**, 99–106.

Davila, D.R., Davis, D.P., Campbell, K., Cambier, J.C., Zigmond, L.A. and Burchiel, S.W. (1995) "Role of alterations in $Ca^{2+}$-associated signaling pathways in the immunotoxicity of polycyclic aromatic hydrocarbons", *Journal of Toxicology and Environmental Health* **45**, 101–126.

Davila, D.R., Romero, D.L. and Burchiel, S.W. (1996) "Human T cells are highly sensitive to suppression of mitogenesis by polycyclic aromatic hydrocarbons and this effect is differentially reversed by alpha-naphthoflavone", *Toxicology and Applied Pharmacology* **139**, 333–341.

de Swart, R.L., Kluten, R.M.G., Huizing, C.J., Vedder, L.J., Reijnders, P.J.H., Visser, I.K.G., Uyt de Haag, F.G.C.M. and Osterhaus, A.D.M.E. (1993) "Mitogen and antigen induced B and T cell responses of peripheral blood mononuclear cells from the harbour seal (*Phoca vitulina*)", *Veterinary Immunology and Immunopathology* **37**, 217–230.

de Swart, R.L., Ross, P.S., Timmerman, H.H., Vos, H.W., Reijnders, P.J.H., Vos, J.G. and Osterhaus, A.D.M.E. (1995) "Impaired cellular immune response in harbor seals (*Phoca vitulina*) feeding on environmentally contaminated herring", *Clinical and Experimental Immunology* **101**, 480–486.

Dietz, R., Heide-Jorgensen, M.-P. and Harkonen, T. (1989) "Mass deaths of harbor seals (*Phoca vitulina*) in Europe", *Ambio* **18**, 258–264.

Di Molfetto-Landon, L., Erickson, K.L., Blanchard-Channell, M., Jeffries, S.J., Harvey, J.T., Jessup, D.A., Ferrick, D.A. and Stott, J.L. (1995) "Blastogenesis and interleukin-2 receptor expression assays in the harbor seal (*Phoca vitulina*)", *Journal of Wildlife Diseases* **31**, 150–158.

Duignan, P.J., Saliki, J.T., St. Aubin, D.J., Early, G., Sadove, S., House, J.A., Kovacs, K. and Geraci, J.R. (1995) "Epizootiology of morbillivirus infection in North American harbor seals (*Phoca vitulina*) and gray seals (*Halichoerus grypus*)", *Journal of Wildlife Diseases* **31**, 491–501.

Fossi, M.C., Marsili, L., Junin, M., Castello, H., Lorenzani, J.A., Casini, S., Savelli, C. and Leonzio, C. (1997) "Use of nondestructive biomarkers and residue analysis to assess the health status of endangered species of pinnipeds in the south-west Atlantic", *Marine Pollution Bulletin* **34**, 163–170.

Geraci, J.R., St. Aubin, D.J., Barker, I.K., Webster, R.G., Hinshaw, V.S., Bean, W.J., Ruhnke, H.R., Prescott, J.H., Early, G., Baker, A.S., Madoff, S. and Schooley, R.T. (1982) "Mass mortality of harbor seals: pneumonia associated with influenza A virus", *Science* **215**, 1129–1131.

Hall, A.J., Law, R.J., Wells, D.E., Harwood, J., Ross, H.M., Kennedy, S., Allchin, C.R., Campbell, L.A. and Pomeroy, P.P. (1992) "Organochlorine levels in common seals (*Phoca vitulina*) which were victims and survivors of the 1988 phocine distemper epizootic", *Science of the Total Environment* **115**, 145–162.

Hart, C.A., Stegeman, J.J. and Hahn, M.E. (1996) "Evidence for cytochrome P450 1A and 2B forms in fur seals (*Callorhinus ursinus*)", *Marine Environmental Research* **42**, 304–305.

Jeffries, S.J., Brown, R.F. and Harvey, J.T. (1993) "Techniques for capturing, handling, and marking harbour seals", *Aquatic Mammals* **19.1**, 21–25.

Kennedy, S., Kuiken, T., Jepson, P.D., Deaville, R., Forsyth, M., Barrett, T., van de Bildt, M.W.G., Osterhaus, A.D.M.E., Eybatov, T., Duck, C., Kydyrmanov, A., Mitrofanov, I. and Wilson, S. (2000) "Mass die-off of Caspian seals caused by canine distemper virus", *Emerging Infectious Diseases* **6**, 637–639.

Kopec, D.A. and Harvey, J.T. (1995) Toxic pollutants, health indices, and population dynamics of harbor seals in San Francisco Bay, 1989–1992. Moss Landing Marine Laboratories Technical Publication, 96-4, ISSN 1088–2413.

Ladics, G.S., Kawabata, T.T. and White, K.L., Jr. (1991) "Suppression of the *in vitro* humoral immune response of mouse splenocytes by 7,12-dimethylbenzanthracene metabolites and inhibition of immunosuppression by alpha-naphthoflavone", *Toxicology and Applied Pharmacology* **110**, 31–44.

Ladics, G.S., Kawabata, T.T., Munson, A.E. and White, K.L., Jr (1992) "Metabolism of benzo[a]pyrene by murine splenic cell types", *Toxicology and Applied Pharmacology* **116**, 248–257.

Lahvis, G.P., Wells, R.S., Kuehl, D.W., Stewart, J.L., Rhinehart, H.L. and Via, C.S. (1995) "Decreased lymphocyte responses in free-ranging bottlenose dolphins (*Tursiops truncatus*) are associated with increased concentrations of PCBs and DDT in peripheral blood", *Environmental Health Perspectives* **104**(4), 67–72.

Luster, M.I., Munson, A.E., Thomas, P.T., Holsapple, M.P., Fenters, J.D., White, K.L., Lauer, L.D., Germolec, D.R., Rosenthal, G.J. and Dean, J.H. (1988) "Methods Evaluation. Development of a testing battery to assess chemical-induced immunotoxicity: National Toxicology Program's guidelines for immunotoxicity evaluation in mice", *Fundamental and Applied Toxicology* **10**, 2–19.

Marine Mammal Commission (1999) In: O'Shea, T.J., Reeves, R.R. and Long, A.K., eds, Marine mammals and persistent ocean contaminants, Proceedings of the Marine Mammal Commission Workshop, Keystone, Colorado, 12–15 October 1998 (Marine Mammal Commission, Bethesda, MD).

Martineau, D., de Guise, S., Fournier, M., Shugart, L., Girard, C., Lagace, A. and Beland, P. (1994) "Pathology and toxicology of beluga whales from the St Lawrence Estuary, Quebec, Canada. Past, present and future", *The Science of the Total Environment* **154**, 201–215.

Olsson, M., Karlsson, B. and Ahnland, E. (1994) "Diseases and environmental contaminants in seals from the Baltic and the Swedish west coast", *The Science of the Total Environment* **154**, 217–227.

Osterhaus, A.D.M.E. and Vedder, E.J. (1988) "Identification of virus causing recent seal deaths", *Nature* **335**, 20.

Reijnders, P.J.H. (1994) "Toxicokinetics of chlorobiphenyls and associated physiological responses in marine mammals, with particular reference to their potential for ecotoxicological risk assessment", *The Science of the Total Environment* **154**, 229–236.

Ross, P., de Swart, R., Addison, R., van Loveren, H., Vos, J. and Osterhaus, A. (1996) "Contaminant-induced immunotoxicity in harbour seals: wildlife at risk?", *Toxicology* **112**, 157–169.

Salas, V.M. and Burchiel, S.W. (1998) "Apoptosis in Daudi human B cells in response to benzo[a]pyrene and benzo[a]pyrene-7,8-dihydrodiol", *Toxicology and Applied Pharmacology* **151**, 367–376.

Simms, W., Jeffries, S., Ikonomou, M. and Ross, P.S. (2000) "Contaminant-related disruption of vitamin A dynamics in free-ranging harbor seal (*Phoca vitulina*) pups from British Columbia, Canada, and Washington State, USA", *Environmental Toxicology and Chemistry* **19**, 2844–2849.

Stack, A.S., Altman-Hamamdzic, S., Morris, P.J., London, S.D. and London, L. (1999) "Polychlorinated biphenyl mixtures (Arochlors) inhibit LPS-induced murine splenocyte proliferation *in vitro*", *Toxicology* **139**, 137–154.

Stegeman, J.J., Miller, C.A., Beyer, J., Moore, M.J. and Goksoyr, A. (1998) "Cytochrome P4501A expression and localization in organs of the minke whale (*Balaenoptera acutorostrata*)", *Marine Environmental Research* **46**, 128.

Tabachnick, B.G. and Fidell, L.S. (1989) Using Multivariate Statistics (HarperCollins, New York).

Tucker, A.N., Vore, S.J. and Luster, M.I. (1986) "Suppression of B cell differentiation by 2,3,7,8-tetrachlorodibenzo-*p*-dioxin", *Molecular Pharmacology* **29**, 372–377.

van Loveren, H., Ross, P.S., Osterhaus, A.D.M.E. and Vos, J.G. (2000) "Contaminant-induced immunosuppression and mass mortalities among harbor seals", *Toxicology Letters* **112–113**, 319–324.

Visser, I.K.G., van Bressem, M.F., Barrett, T. and Osterhaus, A.D.M.E. (1993) "Morbillivirus infections in aquatic mammals", *Veterinary Research* **24**, 169–178.

White, Jr., K.L., Kawabata, T.T. and Ladics, G.S. (1994) "Mechanisms of polycyclic aromatic hydrocarbon immunotoxicity", In: Dean, J.H., Luster, M.I., Munson, A.E. and Kimber, I., eds, Immunotoxicology and Immunopharmacology, 2nd ed., pp 123–142.

Yochem, P.K., Stewart, B.S., de Long, R.L. and de Master, D.P. (1987) "Diel haul-out patterns and site fidelity of harbour seals (*Phoca vitulina richardsi*) on San Miguel Island, California, in autumn", *Marine Mammal Science* **3**, 323–332.

Young, D., Becerra, M., Kopec, D. and Echols, S. (1998) "GC/MS analysis of PCB congeners in blood of the harbor seal (*Phoca vitulina*) from San Francisco Bay", *Chemosphere* **37**, 711–733.

